# Study to explore the mechanism to form inclusion complexes of *β*-cyclodextrin with vitamin molecules

**DOI:** 10.1038/srep35764

**Published:** 2016-10-20

**Authors:** Subhadeep Saha, Aditi Roy, Kanak Roy, Mahendra Nath Roy

**Affiliations:** 1 Department of Chemistry, University of North Bengal, Darjeeling, 734013, India

## Abstract

Host–guest inclusion complexes of *β*-cyclodextrin with two vitamins *viz.*, nicotinic acid and ascorbic acid in aqueous medium have been explored by reliable spectroscopic, physicochemical and calorimetric methods as stabilizer, carrier and regulatory releaser of the guest molecules. Job’s plots have been drawn by UV-visible spectroscopy to confirm the 1:1 stoichiometry of the host-guest assembly. Stereo-chemical nature of the inclusion complexes has been explained by 2D NMR spectroscopy. Surface tension and conductivity studies further support the inclusion process. Association constants for the vitamin-*β*-CD inclusion complexes have been calculated by UV-visible spectroscopy using both Benesi–Hildebrand method and non-linear programme, while the thermodynamic parameters have been estimated with the help of van’t Hoff equation. Isothermal titration calorimetric studies have been performed to determine the stoichiometry, association constant and thermodynamic parameters with high accuracy. The outcomes reveal that there is a drop in ΔS^o^, which is overcome by higher negative value of ΔH^o^, making the overall inclusion process thermodynamically favorable. The association constant is found to be higher for ascorbic acid than that for nicotinic acid, which has been explained on the basis of their molecular structures.

Cyclodextrins (CDs) are the cyclic oligosaccharides containing six (*α*-CD), seven (*β*-CD) and eight (*γ*-CD) glucopyranose units, bound by *α*-(1–4) linkages forming a truncated conical structure^1^,^2^. Thus because of their unique structure, *i.e.*, fairly rigid and well-defined hydrophobic cavities and hydrophilic rims having primary and secondary –OH groups (Fig. 1) they are of particular interest in the modern science^3^,^4^. CDs are used for controlled delivery of organic, inorganic, biological and pharmaceutical molecules due to their ability to form inclusion complexes with diverse guest molecules by encapsulating the non-polar part of the guest into its hydrophobic cavity and stabilizing the polar part by the polar rims^5^,^6^. The use of CDs already has a long history in pharmaceuticals, pesticides, foodstuffs etc. for the solubility, bioavailability, safety, stability and as a carrier of the guest molecules^7^,^8^.

CDs have been widely employed as not only excellent receptors for molecular recognition but also excellent building blocks to construct functional materials, where they could be applied to construct stimuli-responsive supramolecular materials^9^. Series of external stimuli, e.g., enzyme activation, light, temperature, changes in pH or redox and competitive binding may be employed to operate the release of guest molecules from the inclusion composites^10^,^11^. Recently cyclodextrin modified nanoparticles are of great interest as these supramolecular macrocycles significantly combines and enhances the characteristics of the entities, such as the electronic, conductance, thermal, fluorescence and catalytic properties expanding their potential applications as nanosensors, drug delivery vehicles and recycling extraction agents^12^. Different sophisticated probes based on semiconductor nanocrystals and other nanoparticles have been designed for this purpose, because of their potential applications in the fabrication of molecular switches, molecular machines, supramolecular polymers, chemosensors, transmembrane channels, molecule-based logic gates and other interesting host−guest systems^13^,^14^,^15^.

In this article the studied two vitamins, *e.g.*, nicotinic acid and ascorbic acid (Fig. 1) are the essential human nutrients with many important functions in biological systems. Nicotinic acid is used to treat hypercholesterolemia and pellagra while its deficiency causes nausea, skin and mouth lesions, anemia, headaches, and tiredness^16^,^17^. On the other hand scurvy, fatigue, depression, and connective tissue defects are the common syndromes caused by deficiency of ascorbic acid^18^,^19^. Thus to protect these important bio-molecules from external effects (*e.g*., oxidation, structural modification etc.) and for their regulatory release, it is crucial to investigate whether these molecules can be encapsulated into the CD molecule and to explore the thermodynamic aspect of the process. Guorong *et al.,* Okazaki *et al.* and Delicado *et al.* showed different interactions of ascorbic acid with CD, while Manzanares *et al.,* Silva *et al.,* Pardave *et al. and* Hu *et al.* indicated the formation of inclusion complexes between ascorbic acid with *β*-CD by different electro and physicochemical methods^20^,^21^,^22^,^23^,^24^,^25^,^26^. On the other hand Terekhova *et al.* demonstrated nicotinic acid-CD interactions by volumetric and heat capacity studies^27^. In this present work the formation of host-guest inclusion complexes of these two vitamins with *β*-CD (the cavity dimension of which is more appropriate than other CDs to encapsulate a great variety of molecules) have been explored particularly towards their formation, stabilization, carrying and controlled release without chemical modification by different dependable methods like 2D ROESY NMR, UV-Vis spectroscopy, surface tension, conductivity and isothermal titration calorimetric studies, which primarily focuses on the encapsulation of the bio-molecules into the cavity of *β*-CD. The stoichiometry, association constants and thermodynamic parameters for the inclusion complexes have been determined to communicate a quantitative data regarding the encapsulation of the vitamins by *β*-CD.

## Result and Discussion

### Job’s plot reveals the stoichiometry of the host-guest inclusion complex

**Figure 2:**
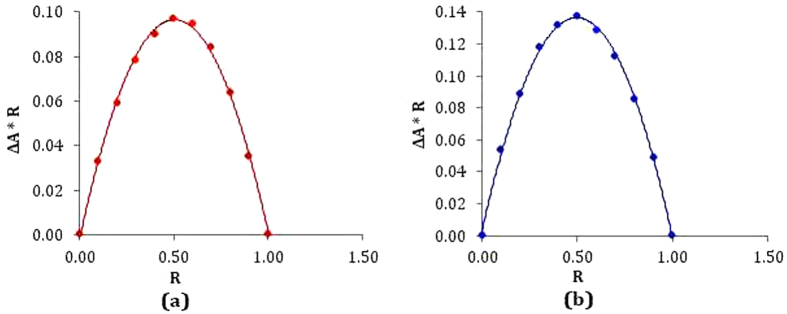
Job’s plot of different vitamin-*β*-CD systems at 298.15 K. (**a**) nicotinic acid at λ_max_ = 260 nm and (**b**) ascorbic acid at λ_max_ = 261 to 265 nm. R = [Vit]/([Vit] + [*β*-CD]), ΔA = absorbance difference of the vitamins without and with *β*-CD.

One of the best methods used to recognize the stoichiometry of the host-guest inclusion complexes is the Job’s method, known as the continuous variation method, which has been applied here by using UV-visible spectroscopy^28^. A set of solutions for each vitamin and β-CD was prepared varying the mole fraction of the guest in the range 0–1 (Tables S1–S2, supplementary data). Job’s plots were generated by plotting ΔA × R against R, where ΔA is the difference in absorbance of the vitamins without and with *β-*CD and R = [Vit]/([Vit] + [β-CD])^29^,^30^. Absorbance values were measured at respective λ_max_ for each solution at 298.15 K. The value of R at the maximum deviation gives the stoichiometry of the inclusion complex (IC), *i.e.,* ratio of guest and host is 1:2 if R = 0.33; 1:1 if R = 0.5; 2:1 if R = 0.66 etc. In the present work maxima for each plot was found at R = 0.5, which suggest 1:1 stoichiometry of the host-guest inclusion complexes (Fig. 2).

### 2D NMR spectra analysis

**Figure 3:**
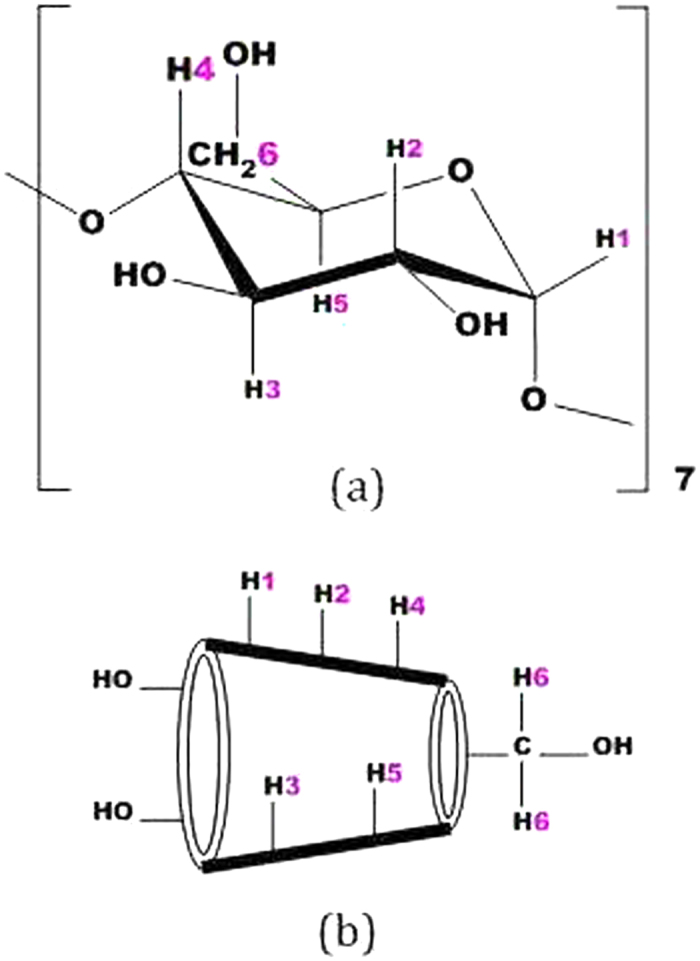
(**a**) Stereo-chemical configuration of *β*-cyclodextrin, (**b**) truncated conical structure of *β*-cyclodextrin with interior and exterior protons.

**Figure 4:**
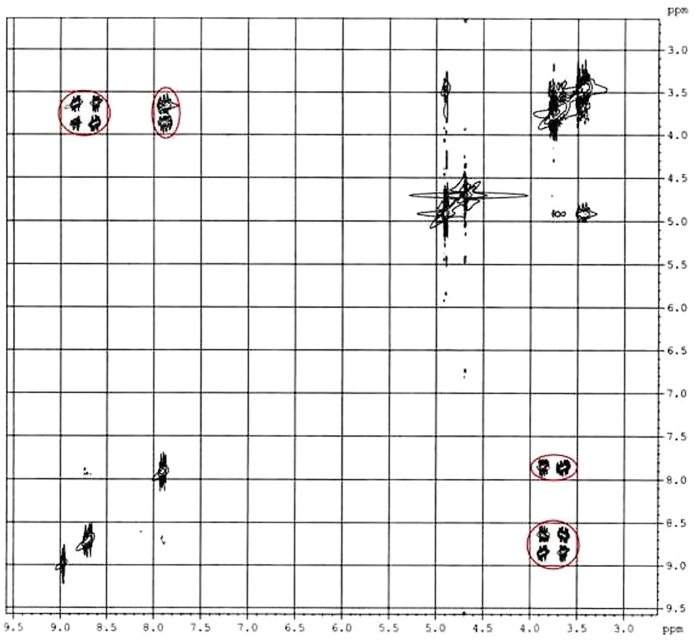
2D ROESY spectra of 1:1 molar ratio of *β*-CD and nicotinic acid in D_2_O (correlation signals are marked by red circles).

**Figure 5:**
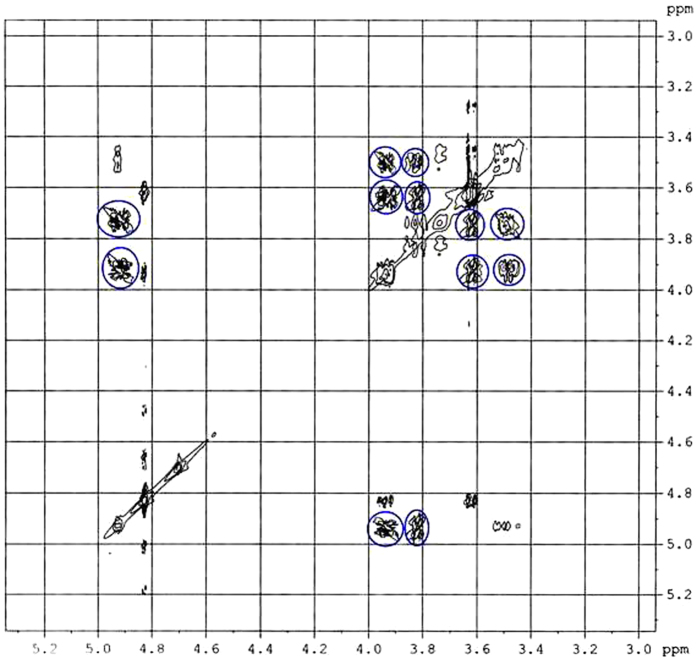
2D ROESY spectra of 1:1 molar ratio of *β*-CD and ascorbic acid in D_2_O (correlation signals are marked by blue circles).

**Figure 6:**
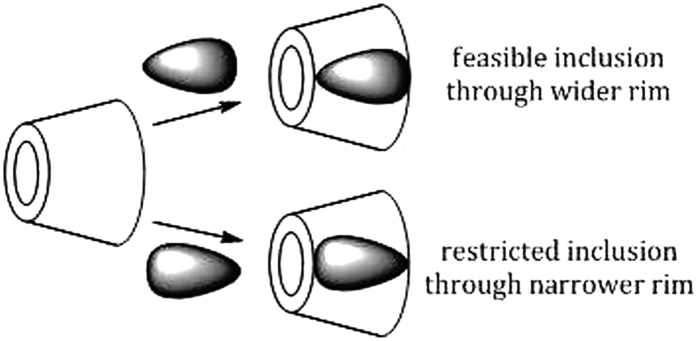
Feasible and restricted inclusion of the guest into the host molecule.

Two-dimensional (2D) NMR spectroscopy gives most powerful evidence about the spatial proximity between the host and the guest atoms by observations of the intermolecular dipolar cross-correlations^31^,^32^. Any two protons that are located within 0.4 nm in space can produce a Nuclear Overhauser Effect (NOE) cross-correlation in NOE spectroscopy (NOESY) or rotating-frame NOE spectroscopy (ROESY)^33^,^34^. In the structure of β-CD the H3 and H5 protons are situated inside the conical cavity, particularly, the H3 are placed near the wider rim while H5 are placed near the narrower rim, the other H1, H2 and H4 protons are located at the exterior of the β-CD molecule (Fig. 3)^35^,^36^. Thus the inclusion phenomenon within the cyclodextrin cavity may be confirmed by the appearance of NOE cross-peaks between the H3 or H5 protons of the host and the protons of the guest identifying their spatial contacts^37^,^38^. For this purpose, 2D ROESY have been obtained of the 1:1 molar mixture of nicotinic acid or ascorbic acid with β-CD. The ROESY spectra of nicotinic acid-β-CD mixture in D_2_O shows appreciable correlation of aromatic protons of nicotinic acid with the H-3 and H-5 protons of β-CD, indicating the aromatic ring was incorporated inside the β-CD cavity (Figs 4, S1 and S2). The ROESY spectra of ascorbic acid-β-CD mixture in D_2_O also shows significant correlations between the H-3, H-5 protons of β-CD and the CH_2_, CHOD, CH protons of ascorbic acid (Figs 5, S3 and S4). This result confirms the encapsulation of the ascorbic acid molecule within the cavity of β-CD. Here in addition the H6 protons of β-CD were not affected by the inclusion process, which tell that the guest vitamin molecules were included into the β-CD cavity via the wider rim (Fig. 6)^39^.

### pH study confirms the ionic states of the vitamins

Measurement of pH of the studied solutions provides important clue about the states of the vitamin molecules in aqueous solution^40^,^41^. It was found that pH of nicotinic acid and ascorbic acid in presence of *β*-CD solution was in the range of 3.35 to 3.46 and 2.92 to 3.16 respectively under experimental condition (Tables S3 and S4). These pH values clearly indicate that both the vitamin molecules release H^+^ ion and exist in the anionic form in aqueous solutions.

### Surface tension study elucidates the inclusion as well as stoichiometric ratio of the host and guest

**Figure 7:**
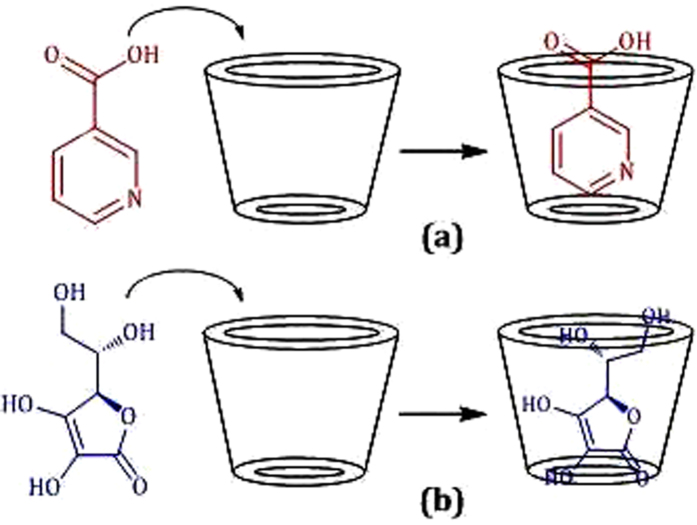
Formation of inclusion complexes of (**a**) nicotinic acid and (**b**) ascorbic acid with *β*-CD.

**Figure 8:**
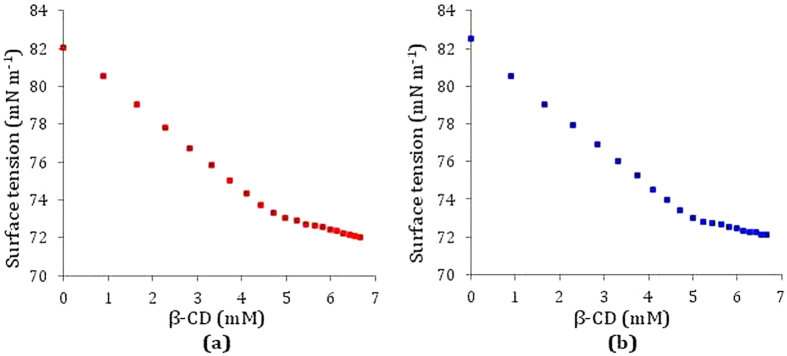
Variation of surface tension of aqueous (**a**) nicotinic acid solution and (**b**) ascorbic acid solution respectively with increasing concentration of *β*-cyclodextrin at 298.15 K.

Surface tension (γ) study gives important clue about the formation and the stoichiometry of the host-guest IC^40^,^41^,^42^. The pH data of aqueous nicotinic acid and ascorbic acid indicate that both the vitamin molecules exist in anionic form, thus, because of ionic interactions there were significant increase in γ of their aqueous solutions. β-CD, in contrast, because of having hydrophobic outer surface and hydrophilic rims, hardly show any change in γ while dissolved in aqueous medium for a wide range of concentration^42^. In the present study γ of aqueous vitamins has been measured with increasing concentration of β-CD at 298.15 K (Tables S3 and S4). Both the vitamins showed progressively falling trend of γ with increasing concentration of β-CD, may be due to encapsulation of the vitamin molecules from the surface of the solution into the hydrophobic cavity of β-CD forming host-guest inclusion complexes (Fig. 7)^43^. Both the plots also show that there are single discernible breaks in each curve, which not only point out the formation of IC but also indicate the 1:1 stoichiometric ratio for each of the ICs formed (Fig. 8)^44^,^45^. The values of γ and corresponding concentrations of vitamins and β-CD at each break have been listed in Table 1, which also indicate that at each break point the concentration ratio of host and guest is about 1:1, establishing the formation of 1:1 ICs between the studied vitamins and β-CD^46^,^47^.

### Conductivity study demonstrates inclusion process and their stoichiometric ratio

**Figure 9:**
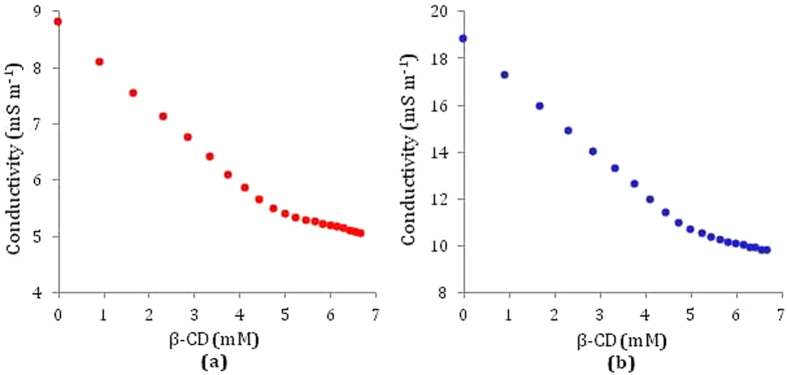
Variation of conductivity of aqueous (**a**) nicotinic acid solution and (**b**) ascorbic acid solution respectively with increasing concentration of *β*-cyclodextrin at 298.15 K.

Conductivity (κ) measurement is an important tool to elucidate the inclusion phenomenon in solution phase^40^,^42^. It indicates the formation as well as the stoichiometry of the IC formed^48^,^49^. In this study the conductivity of the solution decreases gradually as the charged vitamin molecules are encapsulated into the cavity of β-CD, *i.e.*, the conductivity of the solution is markedly affected by the inclusion phenomenon (Tables S3 and S4). At a certain concentration of β-CD and each vitamin a single break was found in each conductivity curve respectively signifying the formation of 1:1 IC (Fig. 9)^40^. The values of κ and corresponding concentrations of the vitamins and β-CD at each break have been listed in Table 2, which reveal that the ratio of the concentrations of each vitamin and β-CD at the break point is approximately 1:1, suggesting that vitamin-cyclodextrin inclusion complex is equimolar, *i.e.*, the host-guest ratio is 1:1 (Fig. 7).

### Ultraviolet spectroscopy: association constants and thermodynamic parameters

Association constants (K_a_) have been calculated for both the vitamin-β-CD ICs by UV-visible spectroscopy. As the vitamin molecules go from the polar aqueous environment to the apolar cavity of β-CD making the IC, there is a change in molar extinction coefficient (∆ε) of the chromophore of the vitamins^50^. The changes in absorbance (∆A) of nicotinic acid (260 nm) and ascorbic acid (261 to 265 nm) were studied against the concentration of β-CD at different temperatures to determine the association constants (K_a_) (Tables S5 and S6). On the basis of reliable Benesi–Hildebrand method for 1:1 host-guest complex the double reciprocal plots have been drawn using Equation 1 (Fig. S5)^30^,^51^.


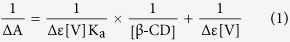


The values of K_a_ for both the systems were evaluated by dividing the intercept by the slope of the straight line of the double reciprocal plot (Table 3)^52^,^53^.

The thermodynamic parameters can easily be derived basing upon the association constants found at various temperatures by the above method with the help of van’t Hoff equation (Equation 2).


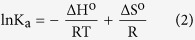


There is a linear relationship between lnK_a_ and 1/T in the above equation (Fig. S6), on the basis of which the thermodynamic parameters ΔH^o^ and ΔS^o^ for the formation of ICs may be obtained (Table S7)^42^,^48^,^54^.

Association constants (K_a_^ψ^) have also been calculated for the vitamin-β-CD ICs by UV-visible spectroscopy with the help of non-linear programme basing upon the changes in absorbance as a result of encapsulation of the vitamin molecule inside into the apolar cavity of β-CD^55^. The following equilibrium is supposed to exist between the host and the guest for 1:1 IC^1^.


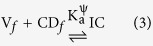


The association constant (K_a_^ψ^) for the formation of IC may be expressed as


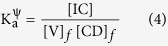


Here, [IC], [V]_*f*_ and [CD]_*f*_ represent the equilibrium concentration of IC, free vitamin molecule and free CD respectively. According to the binding isotherm, the association constant (K_a_^ψ^) for the formation of IC may be expressed as^56^


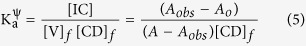


Where,


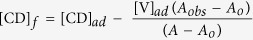


Here, *A*_*o*_, *A*_*obs*_ and *A* are the absorbance of vitamin molecules at initial state, during addition of CD and final state respectively. [V]_*ad*_ and [CD]_*ad*_ are the concentration of vitamin molecule and the added CD respectively. Thus, the values of K_a_^ψ^ for both the systems were evaluated from the binding isotherm by applying non-linear programme (Table 4)^7^,^57^. The corresponding thermodynamic parameters have been derived basing upon the association constants found from various isotherms by the above method with the help of van’t Hoff equation (Equation 2) (Fig. S7, Tables 4 and S8)^48^,^54^.

The values of ΔH^o^ and ΔS^o^ for the formation of ICs were found negative suggesting that the inclusion process is exothermic and entropy controlled but not entropy driven (Table 3)^48^. These results may be explained on the basis of molecular association that was taking place while the ICs were being formed between *β*-CD and each vitamin. Because of these there is a drop of entropy, which is unfavorable for the spontaneity of the IC formation. This effect is conquered by higher negative value of ΔH^o^, making the overall inclusion process thermodynamically favourable.

### Isothermal titration calorimetry: characterization of the complexation

**Figure 10:**
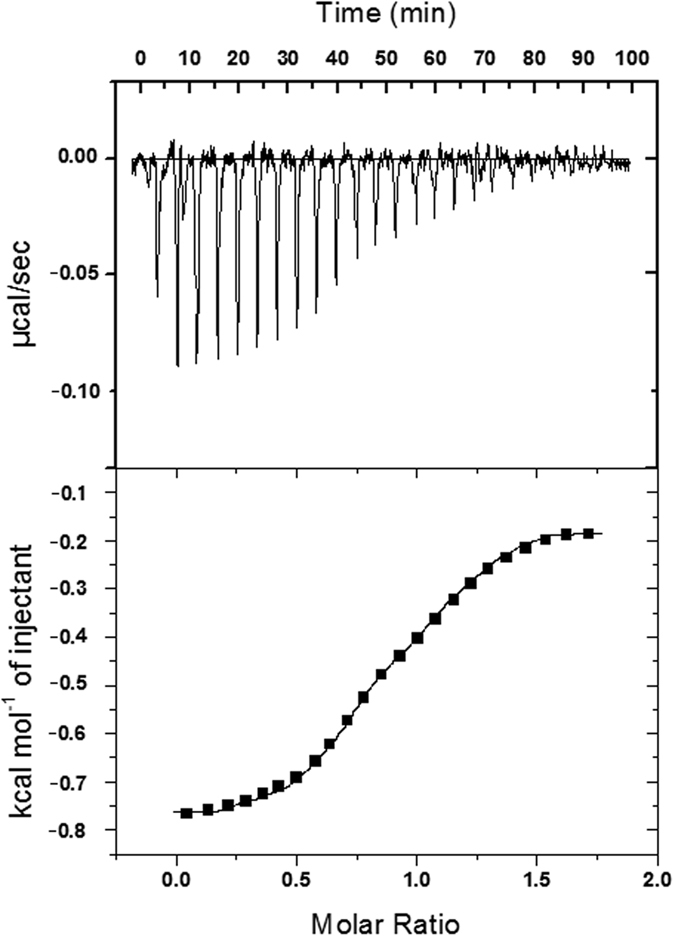
ITC isotherms for the interaction of nicotinic acid with *β*-cyclodextrin at 298 K. For each titration, *β*-cyclodextrin concentration in sample cell was taken as 50 μM and nicotinic acid concentration in syringe was 500 μM. The top panel represents the raw heats of binding obtained upon titration of nicotinic acid to *β*-cyclodextrin. The lower panel is the binding isotherm fitted to the raw data using one site model.

**Figure 11:**
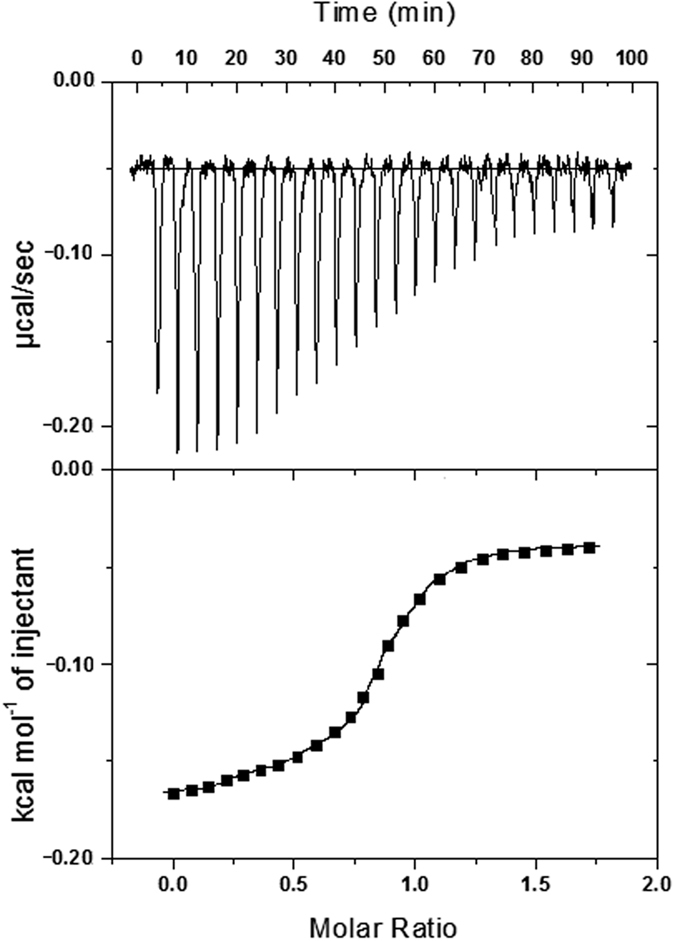
ITC isotherms for the interaction of ascorbic acid with *β*-cyclodextrin at 298 K. For each titration, *β*-cyclodextrin concentration in sample cell was taken as 50 μM and ascorbic acid concentration in syringe was 500 μM. The top panel represents the raw heats of binding obtained upon titration of ascorbic acid to *β*-cyclodextrin. The lower panel is the binding isotherm fitted to the raw data using one site model.

Isothermal titration calorimetry (ITC) is the most sensitive and accurate analytical technique for determination of binding constant and various thermodynamic parameters in host–guest complexation with precise accuracy^58^. It has become an efficient method for direct determination of the thermodynamic parameters rather than using the earlier van’t Hoff equation technique^59^. Top of Fig. 10 shows the data obtained from the ITC titration of NA with β-CD in water at 298 K, which describes production of exothermic heat after each injection and the magnitude of the released heat decreases progressively with each injection until complete complexation is achieved. Bottom of Fig. 10 shows the experimental data and the calculated best fit binding curve of NA with β-CD, that provides the stoichiometry (N^C^), association constant (K_a_^C^), standard enthalpy (ΔH^oC^) and standard entropy (ΔS^oC^) (Table 5). The complexation of AA with β-CD in water at 298 K was similarly studied by ITC as described above for NA-β-CD system and shown in Fig. 11 and the results are listed in Table 5. The outcomes of calorimetric study are consistent with those obtained from the analysis of the UV-visible spectroscopic data, however, these values are little different than those obtained by the earlier spectroscopic method studied at a range of temperature, which may be partly illustrated by the fact that the association constants of CD complexes decrease with increasing temperature, on the basis of which the thermodynamic parameters ∆H^o^ and ∆S^o^ were calculated using van’t Hoff method. But, in calorimetric study these parameters were determined only at 298 K, thus, the variation of the values of association constants is not considered here. The other fact is that in spectroscopic determination, thermodynamic parameters were estimated from association constants, which again were found out on the basis of Δε of the vitamins, that was due to the changes in the environment around the chromophore, when these go from the polar aqueous environment to the apolar cavity of *β*-CD, hence, the changes in enthalpy and entropy described there were exclusively for the formation of IC, not for the other solvent interactions taking place in the medium. But, in calorimetric determination various types of non-covalent forces, like, electrostatic, hydrophobic, van der Waals, and H-bonding are involved in the host–guest interaction, thus, thermodynamic parameters represents an overall heat changes resulting from the above interactions^10^,^60^. Several mechanisms have been proposed for the complexation, where the most important forces involved are van der Waals and hydrophobic interactions^61^. The bindings of NA or AA with β-CD are enthalpy driven as the entropy values of the interactions are not favorable. This indicates electrostatic and hydrophobic interactions play major role in the complexation in these cases.

The stoichiometries (N) of the association further suggest that only 1:1 complexation have occurred in the formation of complexes of NA and AA with β-CD which is in agreement with the 1:1 complexation revealed from the Job’s method.

Formation of the host-guest IC is the dimensional suitability between the two species, which is favored by the unique cyclodextrin molecule that provides an appropriate condition by encapsulating the apolar part of the guest molecule inside the cavity, as well as stabilizing the polar part by the polar rims^47^. The other driving force for the formation of IC is the release of the water molecules from the hydrophobic cavity into the bulk thereby increasing the entropy of the system^1^,^62^. The inclusion of the guest molecule is likely from the wider rim of the *β*-CD molecule to make maximum contact with the cavity (Fig. 6), which is also supported by ROESY spectrum. The polar –OH and –COOH groups of the vitamins can make H-bonds with the –OH groups at both the rims of the *β*-CD molecule. The data shown in Table 3 indicate that K_a_ is greater for ascorbic acid than for nicotinic acid, which is on account of the difference in their structures, *i.e.*, ascorbic acid, because of having more number of –OH groups, makes stronger association at both rims, while nicotinic acid makes H-bonds at the wider rim only (Fig. 7).

## Experimental

### Materials

Nicotinic acid, ascorbic acid and *β*-cyclodextrin of puriss grade were bought from Sigma-Aldrich, Germany and used as purchased. The mass fraction purity of nicotinic acid, ascorbic acid and *β*-cyclodextrin were ≥ 0.99, ≥ 0.99 and ≥0.98 respectively.

### Apparatus and procedure

Prior to the start of the experimental work solubility of *β*-cyclodextrin and chosen vitamins have been precisely checked in triply distilled and degassed water (with a specific conductance of 1 × 10^−6^ S cm^−1^) and observed that the selected vitamins were freely soluble in all proportion of aqueous *β-*cyclodextrin. All the stock solutions of the vitamins were prepared by mass (weighed by Mettler Toledo AG-285 with uncertainty 0.0003 g), and then the working solutions were obtained by mass dilution at 298.15 K. Adequate precautions were made to reduce evaporation loss during mixing.

UV-visible spectra were recorded by JASCO V-530 UV/VIS Spectrophotometer, with an uncertainty of wavelength resolution of ±2 nm. The measuring temperature was held constant by an automated digital thermostat.

2D ROESY spectra were recorded in D_2_O at 300 MHz using Bruker Avance 300 MHz instrument at 298 K. The details of the experiment have been shown in Figs S1 and S3.

pH values of the experimental solutions were measured by Mettler Toledo Seven Multi pH meter with uncertainty ±0.001. The measurements were made in a thermostated water bath maintaining the temperature at 298.15 K. The uncertainty in temperature was ±0.01 K.

The surface tension experiments were done by platinum ring detachment method using a Tensiometer (K9, KRŰSS; Germany) at the experimental temperature. The accuracy of the measurement was within ±0.1 mN m^−1^. Temperature of the system has been maintained by circulating auto-thermostat water through a double-wall glass vessel containing the solution.

Specific conductance values of the experimental solutions were measured by Mettler Toledo Seven Multi conductivity meter with uncertainty ±1.0 *μ*S m^−1^. The measurements were made in an auto-thermostated water bath maintaining the temperature at 298.15 K and using the HPLC grade water with specific conductance of 6.0 *μ*S m^−1^. The cell was calibrated using a 0.01 M aqueous KCl solution. The uncertainty in temperature was ±0.01 K.

Isothermal titration calorimetry was used to obtain association constant at 298 K using a MicroCal VP-ITC (MicroCal, Inc., Northampton, MA, USA). The thermal equilibration step at 298 K was followed by an initial 120 s delay step and the subsequent twenty five injections of each vitamin to β-CD (injection duration of 10 s and spacing of 180 s). Each injection generated a heat-burst curve between micro cal s^−1^ versus time (min). The saturation curve between kcal/mol of injectant vs. molar ratio was determined by integration, using Origin 7.0 software (Microcal, Inc.) to give the measure of the heat associated with the injection. The binding affinity and thermodynamic parameters of the binding process were obtained by fitting the integrated heats of binding the isotherm to the one site binding model to give the association constant (K_a_^C^), stoichiometry (N^C^), binding enthalpy (ΔH°^C^) and the entropy (ΔS°^C^).

## Conclusion

The present study explains that nicotinic acid and ascorbic acid form ICs with *β*-CD in aqueous medium, which can be used as regulatory releaser of the above two vitamins. 2D ROESY NMR study confirms the inclusion phenomenon and its mechanism. Surface tension and conductivity studies also show that the ICs have been formed, the stoichiometry of which were confirmed as 1:1 by Job’s plots. The association constants and thermodynamic parameters have been estimated for both the ICs by reliable spectroscopic and calorimetric techniques with high accuracy, which inform that ascorbic acid-*β*-CD has higher order of association than that of nicotinic acid-*β*-CD. Thus, this work communicates both qualitative and quantitative idea about the formation of ICs of *β*-CD with above two vitamins suggesting their potential applications in pharmaceutical industries and medical sciences.

## Additional Information

**How to cite this article**: Saha, S. *et al.* Study to explore the mechanism to form inclusion complexes of *β*-cyclodextrin with vitamin molecules. *Sci. Rep.* **6**, 35764; doi: 10.1038/srep35764 (2016).

## Supplementary Material

Supplementary Information

## Figures and Tables

**Figure 1:**
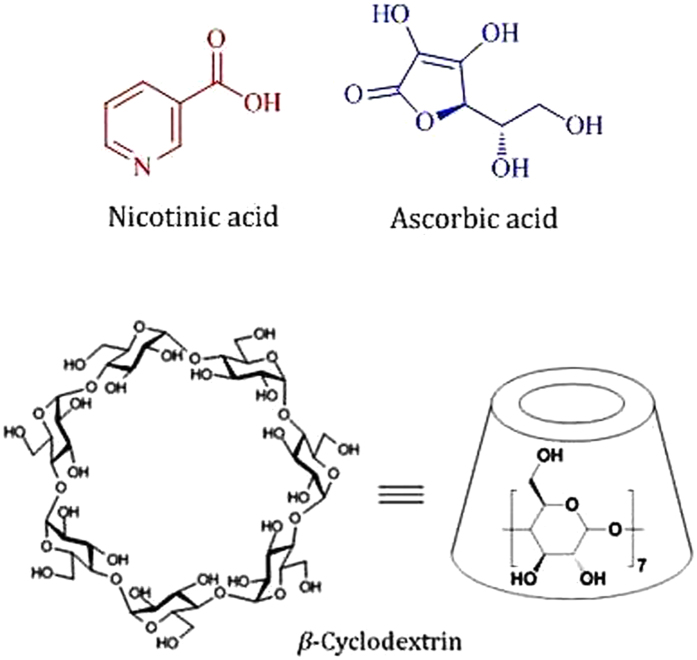
Molecular structure of nicotinic acid, ascorbic acid and *β*-cyclodextrin.

**Table 1:** Values of surface tension (*γ*) at the break point with corresponding concentrations of β-CD and vitamins at 298.15 K^a^.

	Conc of β-CD/mM	Conc of Vit/mM	*γ*^a^/mN·m^−1^
Nicotinic acid	4.81	5.19	73.13
Ascorbic acid	4.94	5.06	72.98

^a^Standard uncertainties (*u*): temperature: *u*(T) = ±0.01 K, surface tension: *u*(*γ*) = ±0.1 mN∙m^−1^.

**Table 2:** Values of conductivity (*κ*) at the break point with corresponding concentrations of *β*-CD and vitamins at 298.15 K^a^.

	Conc of β-CD/mM	Conc of Vit/mM	κ^a^/mS·m^−1^
Nicotinic acid	4.80	5.20	5.42
Ascorbic acid	4.93	5.07	10.65

^*a*^Standard uncertainties (*u*): temperature: *u*(T) = ±0.01 K, conductivity: *u*(*κ*) = ±0.001 mS·m^−1^.

**Table 3:** Association constant (K_a_) and thermodynamic parameters ΔH^o^ and ΔS^o^ of different vitamin-*β*-cyclodextrin inclusion complexes.

	Temp/K^a^	K_a_ × 10^−3^/M^−1^^b^	ΔH^o^/kJ mol^−1^^b^	ΔS^o^/J mol^−1^K^−1^^b^
Nicotinic acid	288.15	1.62	−20.59	−9.96
	293.15	1.39		
	298.15	1.25		
	303.15	1.07		
	308.15	0.92		
	313.15	0.82		
Ascorbic acid	288.15	4.19	−21.67	−5.87
	293.15	3.58		
	298.15	3.10		
	303.15	2.68		
	308.15	2.33		
	313.15	2.03		

^*a*^Standard uncertainties in temperature *u* are: *u*(T) = ±0.01 K.

^*b*^Mean errors in K_**a**_ = ±0.02 × 10^−3^ M^−1^; ΔH^o^ = ±0.01 kJ mol^−1^; ΔS^o^ = ±0.01 J mol^−1^K^−1^.

**Table 4:** Association constants (K_a_^ψ^) obtained from non-linear programme and the corresponding thermodynamic parameters ΔH^oψ^ and ΔS^oψ^ of different vitamin-β-cyclodextrin inclusion complexes.

	Temp/K^a^	K_a_^ψ^ × 10^−3^/M^−1^^b^	ΔH^oψ^/kJ mol^−1^^b^	ΔS^oψ^/J mol^−1^K^−1^^b^
Nicotinic acid	288.15	1.66	−21.28	−12.23
	293.15	1.43		
	298.15	1.23		
	303.15	1.06		
	308.15	0.93		
	313.15	0.82		
Ascorbic acid	288.15	4.21	−21.83	−6.40
	293.15	3.61		
	298.15	3.06		
	303.15	2.64		
	308.15	2.35		
	313.15	2.03		

^*a*^Standard uncertainties in temperature *u* are: *u*(T) = ±0.01 K.

^*b*^Mean errors in K_**a**_^ψ^ = ±0.01 × 10^−3^ M^−1^; ΔH^oψ^ = ±0.01 kJ mol^−1^; ΔS^oψ^ = ±0.01 J mol^−1^K^−1^.

**Table 5:** Stoichiometry (N^C^), association constant (K_a_^C^), standard enthalpy (ΔH^oC^) and standard entropy (ΔS^oC^) of different vitamin-β-cyclodextrin inclusion complexes obtained from isothermal titration calorimetric study at 298.15 K.

	N^C^/Sites	K_a_^C^ × 10^−3^/M^−1^	ΔH^oC^/kJ mol^−1^	ΔS^oC^/J mol^−1^K^−1^
Nicotinic acid	1.06 ± 0.0466	1.498 ± 0.155	−20.58 ± 1.53	−8.96
Ascorbic acid	0.99 ± 0.0111	3.655 ± 0.335	−22.28 ± 1.06	−5.21
