# Supplementary material for: Study to explore the mechanism to form inclusion complexes of β-cyclodextrin with vitamin molecules
Source: Sci Rep. 2016 Oct 20;6:35764. doi: 10.1038/srep35764 (PMC5071908; doi:10.1038/srep35764)
Supplement: Supplementary Information [file srep35764-s1.pdf]

Study to explore the mechanism to form inclusion complexes of β-cyclodextrin with vitamin molecules

Subhadeep Saha, Aditi Roy, Kanak Roy & Mahendra Nath Roy

Department of Chemistry, University of North Bengal, Darjeeling-734013, India

E-mail: *mahendraroy2002@yahoo.co.in*

Tables:

Table S1 Data for the Job plot performed by UV-Vis spectroscopy for aqueous nicotinic acid-β-CD system at 298.15K[a]

Nicotinic acid (mL)	β-CD (mL)	Nicotinic acid (μM)	β-CD (μM)	[NA]/([NA]+[β-CD])	Absorbance (A)	ΔA	ΔA×[NA]/([NA]+[β-CD])
0.0	1.0	0	100	0.0	0.0000	0.3625	0.0000
0.1	0.9	10	90	0.1	0.0357	0.3268	0.0327
0.2	0.8	20	80	0.2	0.0664	0.2961	0.0592
0.3	0.7	30	70	0.3	0.1022	0.2602	0.0781
0.4	0.6	40	60	0.4	0.1369	0.2256	0.0902
0.5	0.5	50	50	0.5	0.1681	0.1944	0.0972
0.6	0.4	60	40	0.6	0.2044	0.1581	0.0948
0.7	0.3	70	30	0.7	0.2421	0.1204	0.0842
0.8	0.2	80	20	0.8	0.2828	0.0797	0.0638
0.9	0.1	90	10	0.9	0.3233	0.0392	0.0353
1.0	0.0	100	0	1.0	0.3625	0.0000	0.0000

[a] Standard uncertainties in temperature u are: u(T) = ±0.01 K.

Table S2 Data for the Job plot performed by UV-Vis spectroscopy for aqueous ascorbic acid-β-CD system at 298.15K[a]

Ascorbic acid (mL)	β-CD (mL)	Ascorbic acid (μM)	β-CD (μM)	[AA]/([AA]+[β-CD])	Absorbance (A)	ΔA	ΔA×[AA]/([AA]+[β-CD])
0.0	1.0	0	100	0.0	0.0000	0.6877	0.0000
0.1	0.9	10	90	0.1	0.1489	0.5388	0.0539

0.2	0.8	20	80	0.2	0.2460	0.4417	0.0883
0.3	0.7	30	70	0.3	0.2935	0.3942	0.1182
0.4	0.6	40	60	0.4	0.3587	0.3290	0.1316
0.5	0.5	50	50	0.5	0.4134	0.2743	0.1371
0.6	0.4	60	40	0.6	0.4737	0.2139	0.1284
0.7	0.3	70	30	0.7	0.5268	0.1609	0.1126
0.8	0.2	80	20	0.8	0.5811	0.1066	0.0853
0.9	0.1	90	10	0.9	0.6333	0.0543	0.0489
1.0	0.0	100	0	1.0	0.6877	0.0000	0.0000

[a] Standard uncertainties in temperature *u* are: *u*(T) = ±0.01 K.

Table S3 Data for surface tension, conductivity and pH study of aqueous nicotinic acid-*β*-CD system at 298.15K[a]

Volm of *β*-CD (mL)	Total volm (mL)	Conc of Nicotinic acid (mM)	Conc of *β*-CD (mM)	Surface tension (mN m^{-1})	Conductuvity (mS m^{-1})	pH
0	10	10.000	0.000	82.0	8.81	3.349
1	11	9.091	0.909	80.5	8.10	3.359
2	12	8.333	1.667	79.0	7.55	3.367
3	13	7.692	2.308	77.8	7.13	3.377
4	14	7.143	2.857	76.7	6.75	3.385
5	15	6.667	3.333	75.8	6.41	3.393
6	16	6.250	3.750	75.0	6.10	3.401
7	17	5.882	4.118	74.3	5.85	3.409
8	18	5.556	4.444	73.7	5.65	3.416
9	19	5.263	4.737	73.3	5.49	3.423
10	20	5.000	5.000	73.0	5.39	3.428
11	21	4.762	5.238	72.9	5.32	3.432
12	22	4.545	5.455	72.7	5.29	3.436
13	23	4.348	5.652	72.6	5.26	3.440
14	24	4.167	5.833	72.5	5.22	3.442
15	25	4.000	6.000	72.4	5.19	3.445
16	26	3.846	6.154	72.3	5.17	3.447
17	27	3.704	6.296	72.2	5.14	3.450

18	28	3.571	6.429	72.1	5.11	3.453
19	29	3.448	6.552	72.1	5.08	3.455
20	30	3.333	6.667	72.0	5.06	3.458

[a] Standard uncertainties in temperature *u* are: *u*(T) = ±0.01 K.

Table S4 Data for surface tension, conductivity and pH study of aqueous ascorbic acid-*β*-CD system at 298.15K[a]

Volm of *β*-CD (mL)	Total volm (mL)	Conc of Ascorbic acid (mM)	Conc of *β*-CD (mM)	Surface tension (mN m^{-1})	Conductuvity (mS m^{-1})	pH
0	10	10.000	0.000	82.5	18.85	2.916
1	11	9.091	0.909	80.5	17.25	2.921
2	12	8.333	1.667	79.0	15.94	2.935
3	13	7.692	2.308	77.9	14.90	2.955
4	14	7.143	2.857	76.9	14.00	2.970
5	15	6.667	3.333	76.0	13.29	2.986
6	16	6.250	3.750	75.2	12.62	3.002
7	17	5.882	4.118	74.5	11.98	3.020
8	18	5.556	4.444	73.9	11.40	3.031
9	19	5.263	4.737	73.4	10.95	3.045
10	20	5.000	5.000	73.0	10.68	3.056
11	21	4.762	5.238	72.8	10.50	3.067
12	22	4.545	5.455	72.7	10.35	3.081
13	23	4.348	5.652	72.6	10.25	3.092
14	24	4.167	5.833	72.5	10.16	3.104
15	25	4.000	6.000	72.4	10.07	3.115
16	26	3.846	6.154	72.3	10.01	3.123
17	27	3.704	6.296	72.2	9.94	3.136
18	28	3.571	6.429	72.2	9.90	3.145
19	29	3.448	6.552	72.1	9.83	3.154
20	30	3.333	6.667	72.1	9.80	3.162

[a] Standard uncertainties in temperature *u* are: *u*(T) = ±0.01 K.

Table S5 Data for the Benesi-Hildebrand double reciprocal plot performed by UV-Vis spectroscopy for aqueous nicotinic acid-β-CD system

Temp /K[a]	[NA] /μM	[β-CD] /μM	Ao	A	ΔA	1/[β-CD] /M^{-1}	1/ΔA	Intercept	Slope	K_a /M^{-1}
	50	30		0.1793	0.0073	33333	137.0			
	50	40		0.1818	0.0098	25000	102.0			
288.15	50	50	0.1720	0.1839	0.0119	20000	84.0	6.2982651	0.0038934	1617.68
	50	60		0.1860	0.0140	16667	71.4			
	50	70		0.1880	0.0160	14286	62.5			
	50	30		0.1784	0.0064	33333	156.3			
	50	40		0.1805	0.0085	25000	117.6			
293.15	50	50	0.1720	0.1824	0.0104	20000	96.2	6.2545665	0.0044882	1393.56
	50	60		0.1843	0.0123	16667	81.3			
	50	70		0.1862	0.0142	14286	70.4			
	50	30		0.1774	0.0054	33333	185.2			
	50	40		0.1790	0.0070	25000	142.9			
298.15	50	50	0.1720	0.1808	0.0088	20000	113.6	6.7025956	0.0053795	1245.95
	50	60		0.1823	0.0103	16667	97.1			
	50	70		0.1841	0.0121	14286	82.6			
	50	30		0.1769	0.0049	33333	204.1			
	50	40		0.1783	0.0063	25000	158.7			
303.15	50	50	0.1720	0.1799	0.0079	20000	126.6	6.4153885	0.0059772	1073.31
	50	60		0.1816	0.0096	16667	104.2			
	50	70		0.1829	0.0109	14286	91.7			
	50	30		0.1762	0.0042	33333	238.1			
	50	40		0.1777	0.0057	25000	175.4			
308.15	50	50	0.1720	0.1790	0.0070	20000	142.9	6.3315992	0.0068908	918.85
	50	60		0.1802	0.0082	16667	122.0			
	50	70		0.1814	0.0094	14286	106.4			
	50	30		0.1757	0.0037	33333	270.3			
	50	40		0.1769	0.0049	25000	204.1			
313.15	50	50	0.1720	0.1782	0.0062	20000	161.3	6.4667146	0.0078986	818.72
	50	60		0.1791	0.0071	16667	140.8			
	50	70		0.1804	0.0084	14286	119.0			

[a] Standard uncertainties in temperature u are: u(T) = ±0.01 K.

Table S6 Data for the Benesi-Hildebrand double reciprocal plot performed by UV-Vis spectroscopy for aqueous ascorbic acid-β-CD system

Temp /K[a]	[AA] /μM	[β-CD] /μM	Ao	A	ΔA	1/[β-CD] /M^{-1}	1/ΔA	Intercept	Slope	K_a /M^{-1}
	50	30		0.4098	0.0078	33333	128.2			
	50	40		0.4120	0.0100	25000	100.0			
288.15	50	50	0.4020	0.4139	0.0119	20000	84.0	14.350533	0.0034272	4187.25
	50	60		0.4159	0.0139	16667	71.9			
	50	70		0.4181	0.0161	14286	62.1			
	50	30		0.4088	0.0068	33333	147.1			
	50	40		0.4103	0.0083	25000	120.5			
293.15	50	50	0.4020	0.4123	0.0103	20000	97.1	14.545396	0.0040579	3584.46
	50	60		0.4144	0.0124	16667	80.6			
	50	70		0.4161	0.0141	14286	70.9			
	50	30		0.4078	0.0058	33333	172.4			
	50	40		0.4095	0.0075	25000	133.3			
298.15	50	50	0.4020	0.4112	0.0092	20000	108.7	14.671623	0.0047343	3099.01
	50	60		0.4126	0.0106	16667	94.3			
	50	70		0.4142	0.0122	14286	82.0			
	50	30		0.4071	0.0051	33333	196.1			
	50	40		0.4085	0.0065	25000	153.8			
303.15	50	50	0.4020	0.4102	0.0082	20000	122.0	14.632896	0.005469	2675.61
	50	60		0.4114	0.0094	16667	106.4			
	50	70		0.4128	0.0108	14286	92.6			
	50	30		0.4065	0.0045	33333	222.2			
	50	40		0.4075	0.0055	25000	181.8			
308.15	50	50	0.4020	0.4090	0.0070	20000	142.9	14.83579	0.0063582	2333.33
	50	60		0.4104	0.0084	16667	119.0			
	50	70		0.4117	0.0097	14286	103.1			
	50	30		0.4057	0.0037	33333	270.3			
	50	40		0.4067	0.0047	25000	212.8			
313.15	50	50	0.4020	0.4079	0.0059	20000	169.5	15.638863	0.0077024	2030.39
	50	60		0.4091	0.0071	16667	140.8			
	50	70		0.4099	0.0079	14286	126.6			

[a] Standard uncertainties in temperature u are: u(T) = ±0.01 K.

Table S7 Data of the van't Hoff equation for calculation of thermodynamic parameters ΔH^o and ΔS^o of different vitamin-β-cyclodextrin inclusion complexes

	Temp /K^a	K_a /M^{-1}	1/T	lnK_a	Intercept	Slope	ΔH^o /kJ mol^{-1}	ΔS^o /J mol^{-1}K^{-1}
Nicotinic acid	288.15	1618	0.00347	7.3887	-1.1985	2476.18	-20.59	-9.96
	293.15	1394	0.00341	7.2396				
	298.15	1246	0.00335	7.1277				
	303.15	1073	0.00330	6.9785				
	308.15	919	0.00325	6.8231				
	313.15	819	0.00319	6.7077				
Ascorbic acid	288.15	4187	0.00347	8.3398	-0.7058	2606.59	-21.67	-5.87
	293.15	3584	0.00341	8.1844				
	298.15	3099	0.00335	8.0388				
	303.15	2676	0.00330	7.8919				
	308.15	2333	0.00325	7.7551				
	313.15	2030	0.00319	7.6160				

[a] Standard uncertainties in temperature *u* are: *u*(T) = ±0.01 K.

Table S8 Data of the van't Hoff equation for calculation of thermodynamic parameters ΔH$^{o\Psi}$ and ΔS$^{o\Psi}$ of different vitamin-β-cyclodextrin inclusion complexes

	Temp /K^a	K_a^{Ψ} /M^{-1}	1/T	lnK_a^{Ψ}	Intercept	Slope	ΔH$^{o\Psi}$ /kJ mol^{-1}	ΔS$^{o\Psi}$ /J mol^{-1}K^{-1}
Nicotinic acid	288.15	1655	0.00347	7.412	-1.4704	2559.29	-21.28	-12.23
	293.15	1425	0.00341	7.262				
	298.15	1229	0.00335	7.114				
	303.15	1059	0.00330	6.965				
	308.15	933	0.00325	6.838				
	313.15	815	0.00319	6.703				
Ascorbic acid	288.15	4209	0.00347	8.345	-0.7695	2625.40	-21.83	-6.40
	293.15	3612	0.00341	8.192				
	298.15	3061	0.00335	8.026				
	303.15	2644	0.00330	7.880				
	308.15	2349	0.00325	7.762				
	313.15	2028	0.00319	7.615				

[a] Standard uncertainties in temperature *u* are: *u*(T) = ±0.01 K.

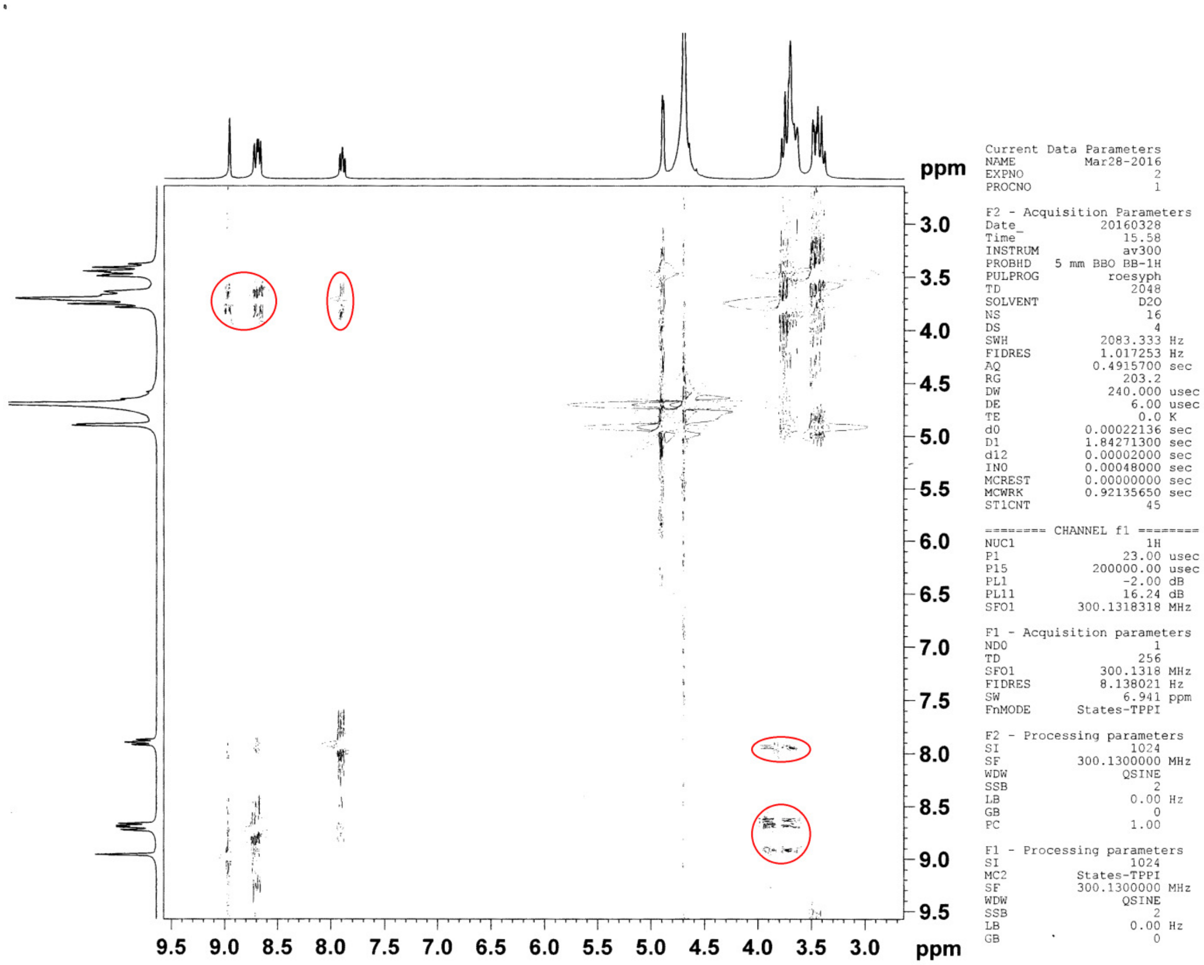

Fig S1. 2D ROESY spectra of 1:1 molar ratio of β-CD and nicotinic acid in D_2O. Red circles showing the correlation signals.

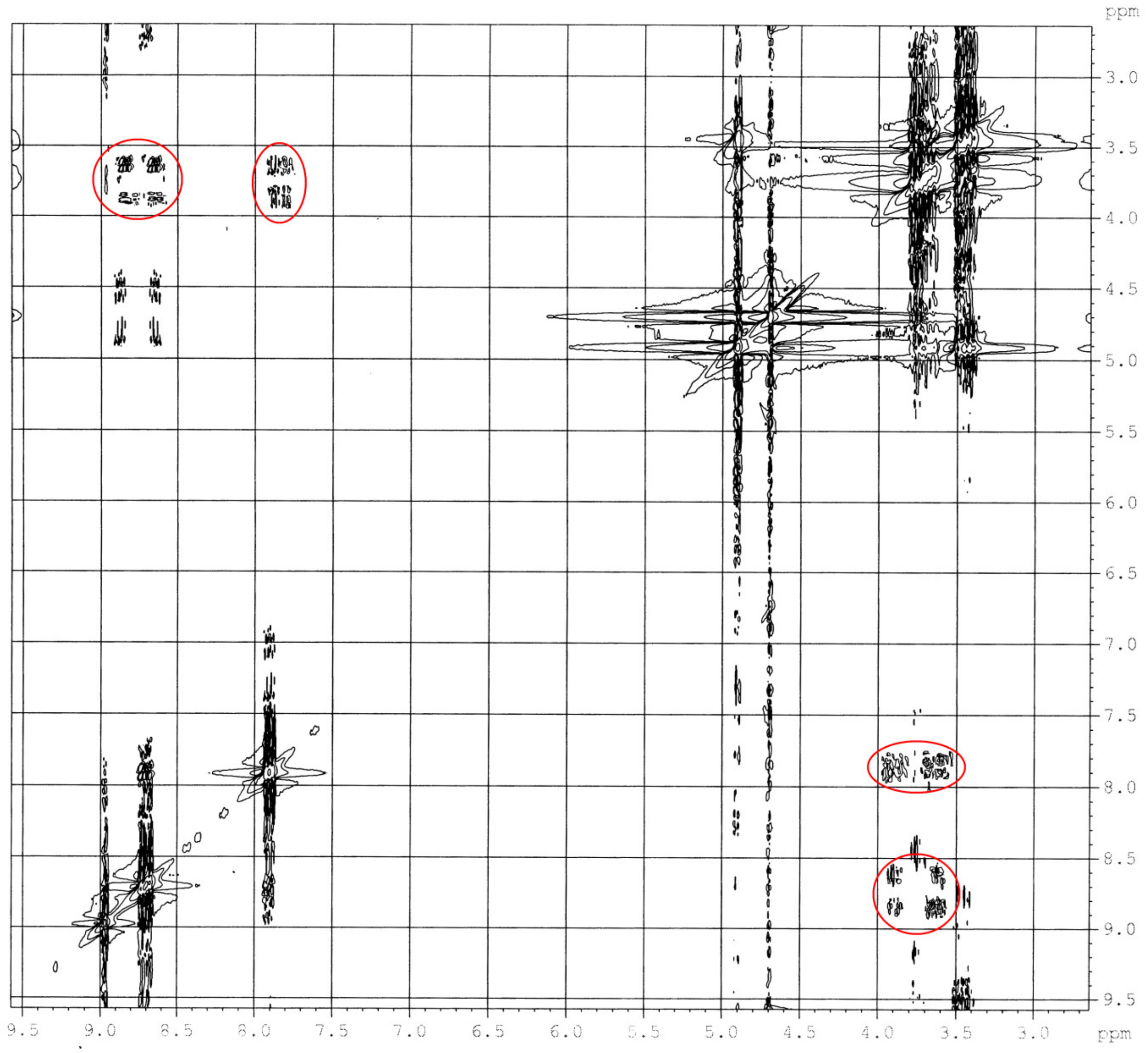

Fig S2. 2D ROESY spectra of 1:1 molar ratio of *β*-CD and nicotinic acid in D_2O. Red circles showing the correlation signals.

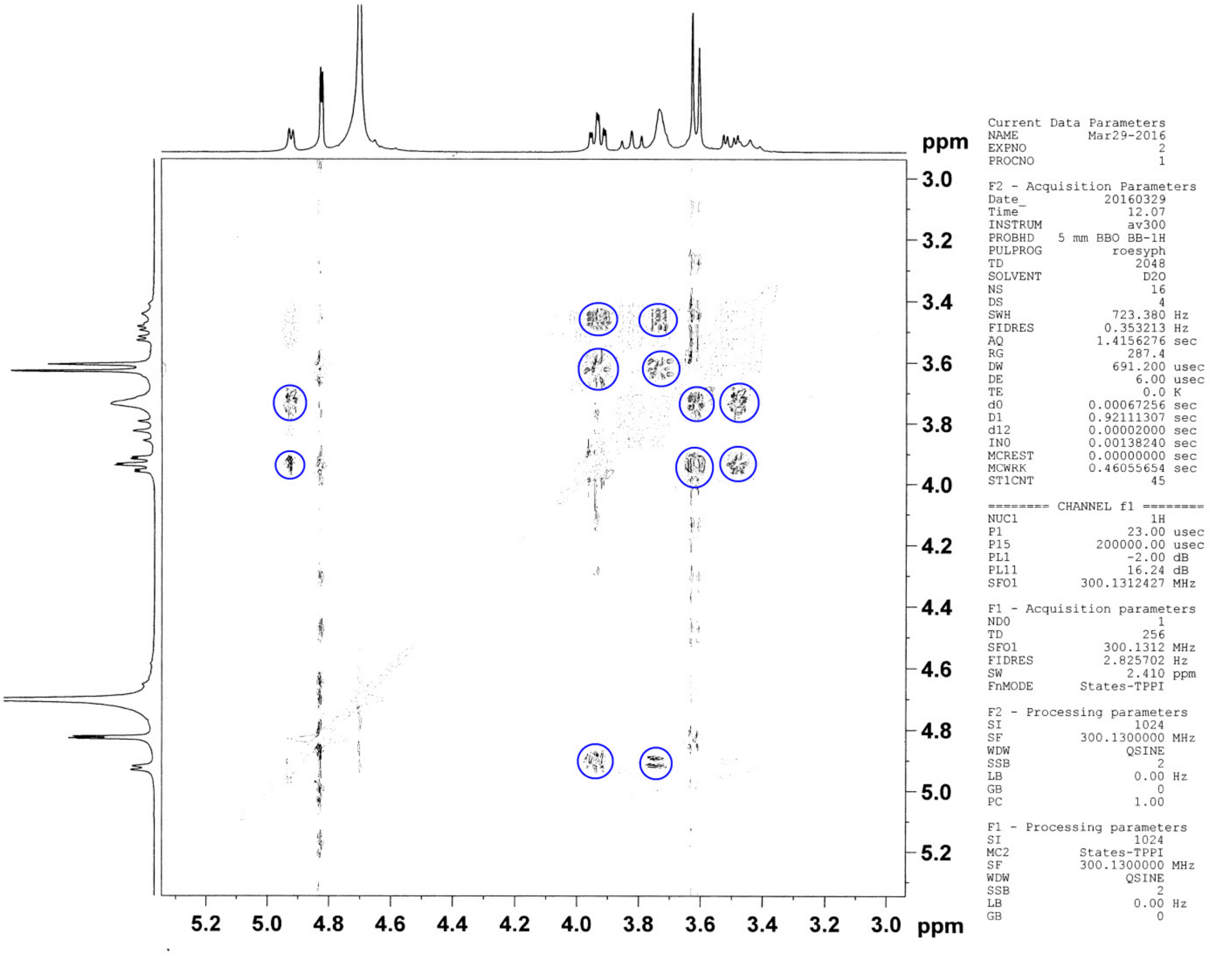

Fig S3. 2D ROESY spectra of 1:1 molar ratio of β-CD and ascorbic acid in D_2O. Blue circles showing the correlation signals.

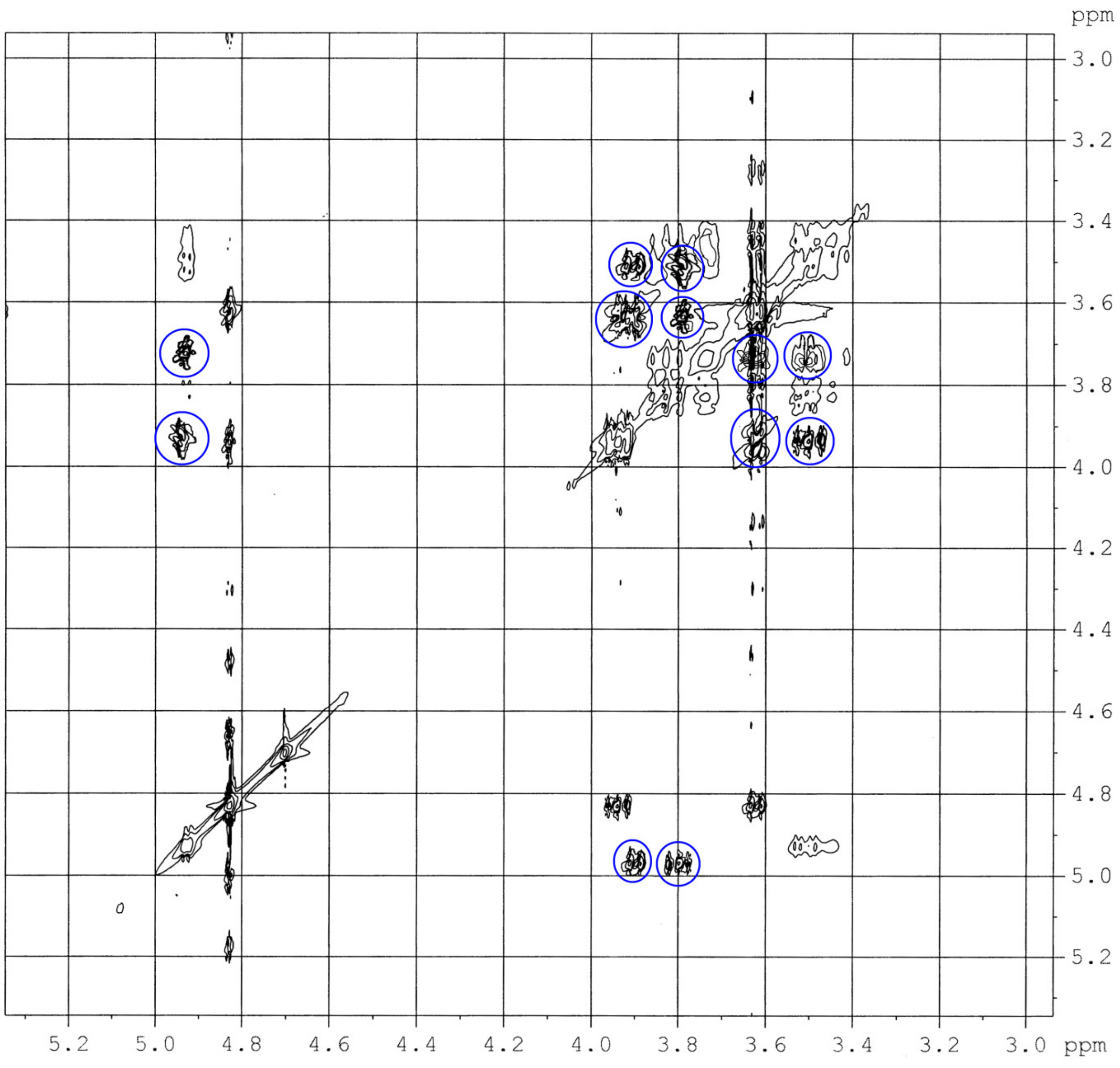

Fig S4. 2D ROESY spectra of 1:1 molar ratio of β-CD and ascorbic acid in D_2O. Blue circles showing the correlation signals.

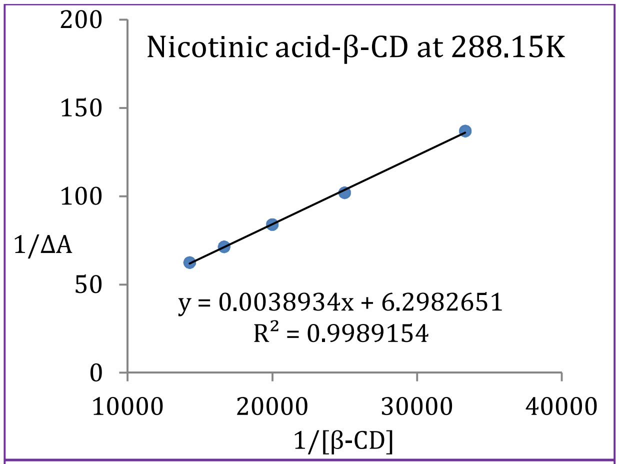
Nicotinic acid-β-CD at 288.15K
y = 0.0038934x + 6.2982651
R² = 0.9989154
1/ΔA
1/[β-CD]

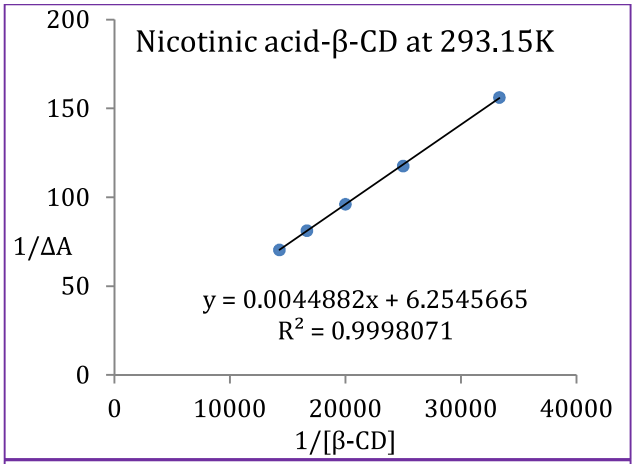
Nicotinic acid-β-CD at 293.15K
y = 0.0044882x + 6.2545665
R² = 0.9998071
1/ΔA
1/[β-CD]

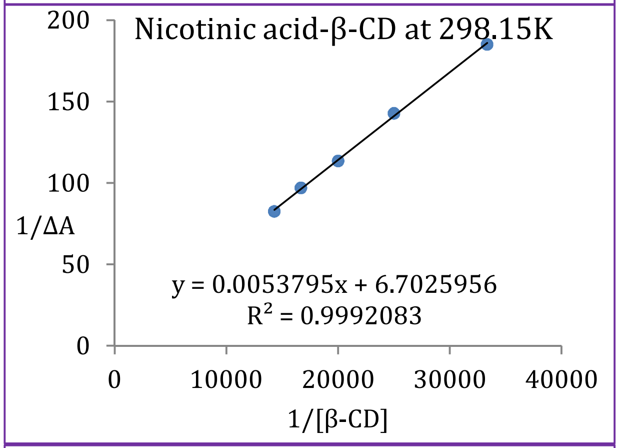
Nicotinic acid-β-CD at 298.15K
y = 0.0053795x + 6.7025956
R² = 0.9992083
1/ΔA
1/[β-CD]

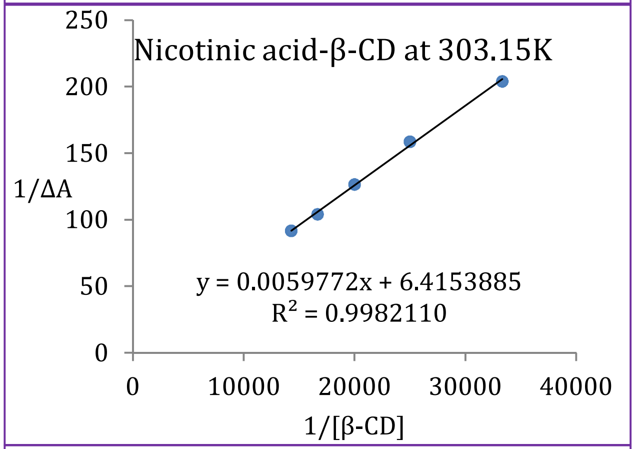
Nicotinic acid-β-CD at 303.15K
y = 0.0059772x + 6.4153885
R² = 0.9982110
1/ΔA
1/[β-CD]

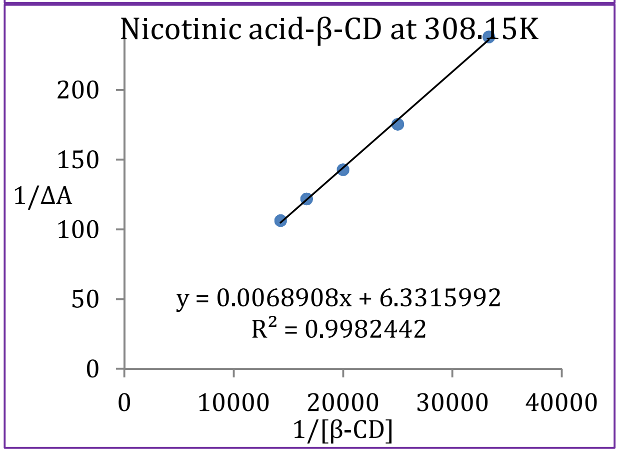
Nicotinic acid-β-CD at 308.15K
y = 0.0068908x + 6.3315992
R² = 0.9982442
1/ΔA
1/[β-CD]

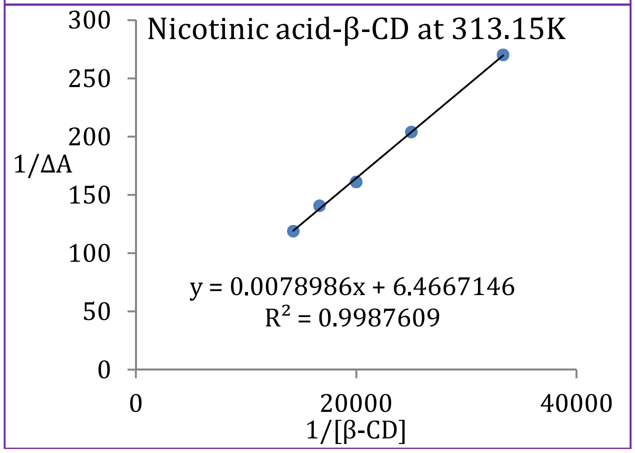
Nicotinic acid-β-CD at 313.15K
y = 0.0078986x + 6.4667146
R² = 0.9987609
1/ΔA
1/[β-CD]

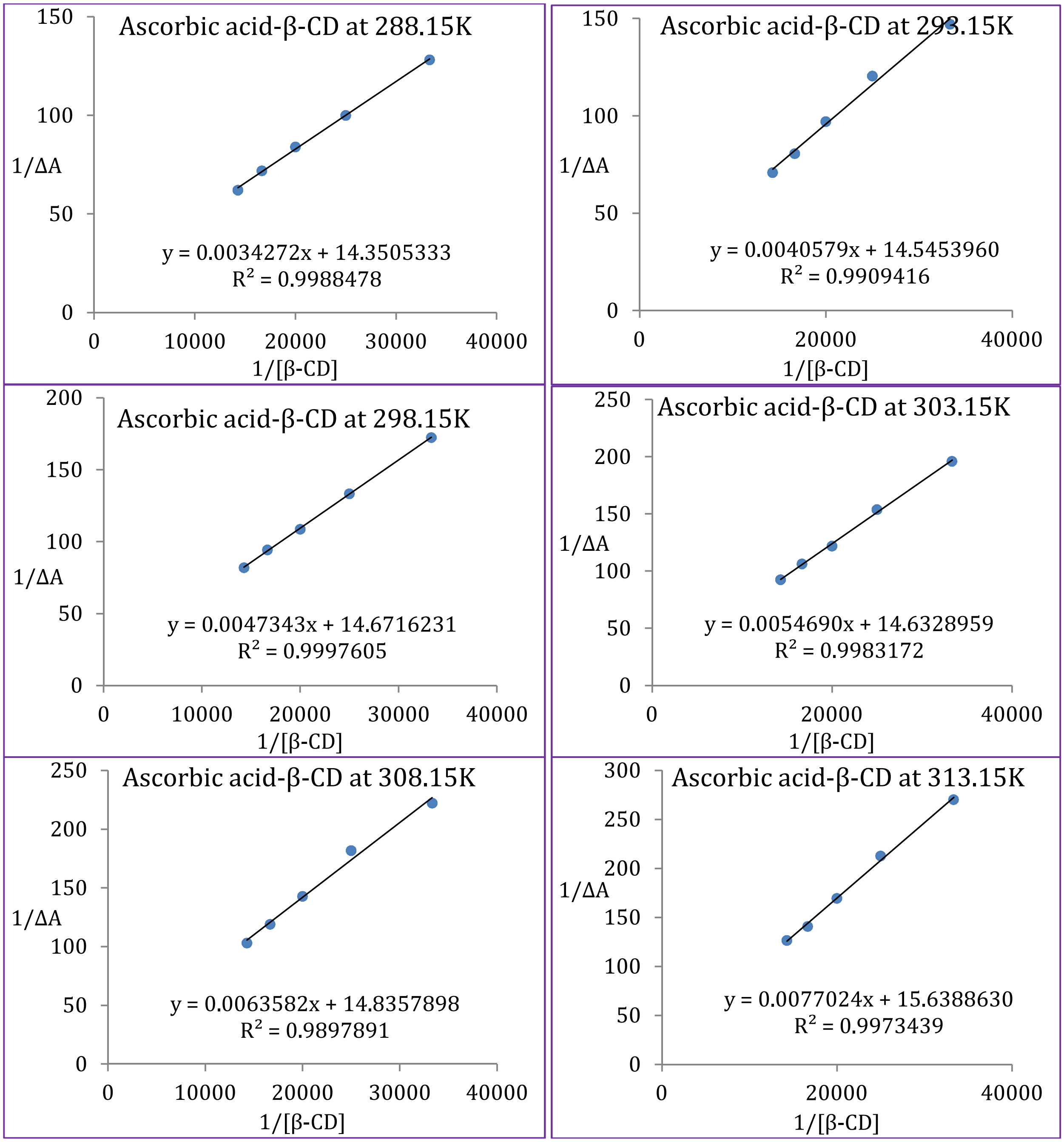

Fig S5. Benesi-Hildebrand double reciprocal plot for the effect of β-CD on the absorbance of nicotinic acid (260 nm) and ascorbic acid (261 to 265 nm) at different temperatures.

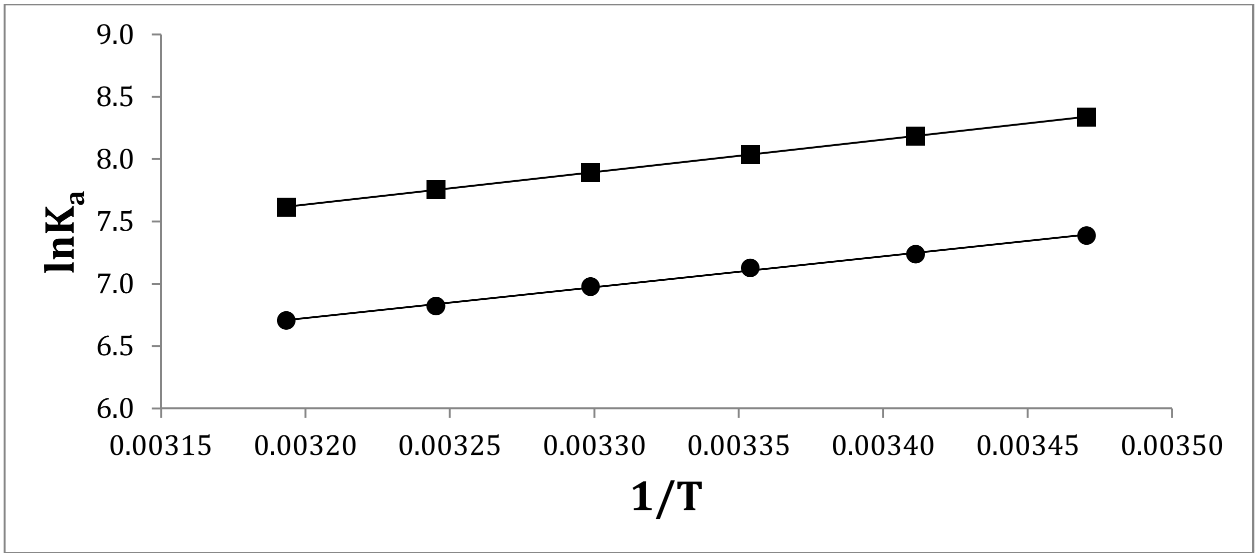

Fig S6. Plot of lnK_a vs 1/T for the interaction between nicotinic acid (●) and ascorbic acid (■) with β-CD.

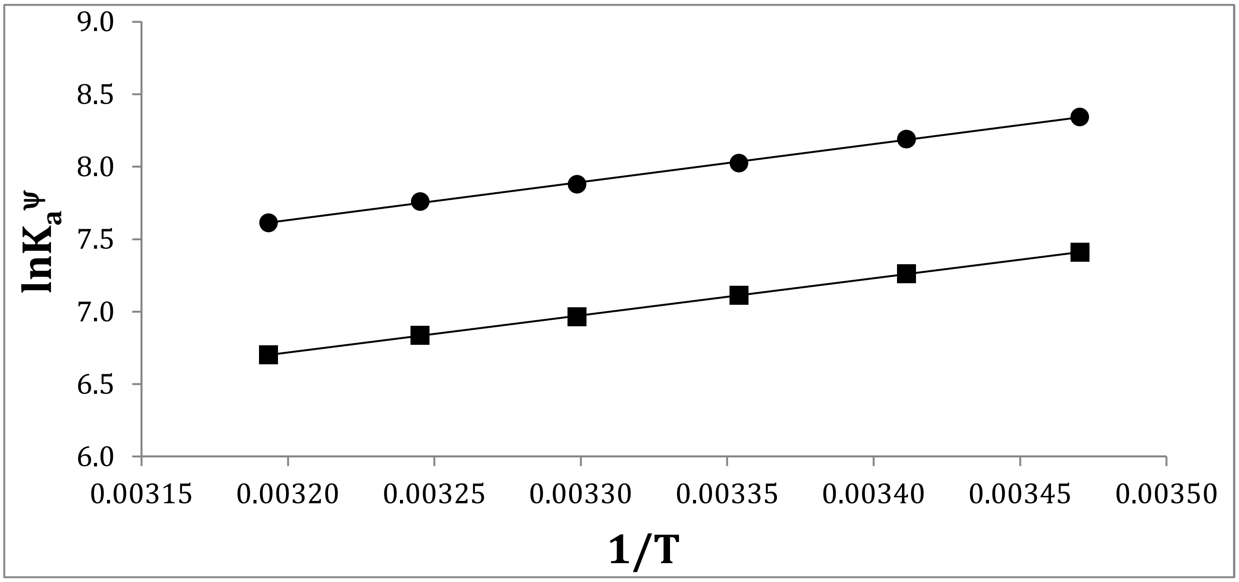

Fig S7. Plot of lnK_a^Ψ vs 1/T for the interaction between nicotinic acid (■) and ascorbic acid (●) with β-CD.
